# Guidelines for treatment of immune-mediated cerebellar ataxias

**DOI:** 10.1186/s40673-015-0034-y

**Published:** 2015-11-10

**Authors:** Hiroshi Mitoma, Marios Hadjivassiliou, Jérôme Honnorat

**Affiliations:** Department of Medical Education, Tokyo Medical University, Tokyo, Japan; Academic Department of Neurosciences, Royal Hallamshire Hospital, Sheffield, UK; University Lyon 1, University Lyon, Rue Guillaume Paradin, 69372 Lyon, Cedex 08 France; INSERM, UMR-S1028, CNRS, UMR-5292, Lyon Neuroscience Research Center, Neuro-Oncology and Neuro-Inflammation Team, 7, Rue Guillaume Paradin, 69372 Lyon, Cedex 08 France; National Reference Centre for Paraneoplastic Neurological Diseases, Hospices civils de Lyon, Hôpital neurologique, 69677 Bron, France; Hospices Civils de Lyon, Neuro-oncology, Hôpital Neurologique, 69677 Bron, France

**Keywords:** Cerebellar ataxias, Gluten ataxia, Paraneoplastic cerebellar degeneration, Hashimoto’s encephalopathy, Anti-GAD antibodies, Immunotherapy

## Abstract

Immune-mediated cerebellar ataxias include gluten ataxia, paraneoplastic cerebellar degeneration, GAD antibody associated cerebellar ataxia, and Hashimoto’s encephalopathy. Despite the identification of an increasing number of immune-mediated cerebellar ataxias, there is no proposed standardized therapy. We evaluated the efficacies of immunotherapies in reported cases using a common scale of daily activity. The analysis highlighted the importance of removal of autoimmune triggering factors (e.g., gluten or cancer) and the need for immunotherapy evaluation (e.g., corticosteroids, intravenous immunoglobulin, immunosuppressants) and adaptation according to each subtype.

**Electronic supplementary material:**

The online version of this article (doi:10.1186/s40673-015-0034-y) contains supplementary material, which is available to authorized users.

## Background

Accumulating evidence suggests that the cerebellum is one of the main CNS targets of autoimmunity, as demonstrated by the high prevalence of paraneoplastic cerebellar degeneration (PCD) amongst paraneoplastic neurological syndromes [1, 2]. Since the first description of PCD in 1919 [3], a variety of autoantibodies have been identified associated with different neoplasms [4]. In addition to the well-established concept of PCD, the clinical entity of non-paraneoplastic immune-mediated cerebellar ataxias (CAs) was established recently [5–7]. In the 1980s, some of these cases were reported in association with autoantibodies, and three clinical entities have been established since then, based on the type of the associated antibodies (Abs) and specific clinical features: gluten ataxia (GA), anti-glutamic acid decarboxylase antibodies (GAD Abs)-associated cerebellar ataxia (GAD Abs-CA), and Hashimoto’s encephalopathy (HE). Other clinical entities will probably emerge in the future because some autoantibodies have been described recently in patients with cerebellar ataxia, but as only few patients have been described in each group, further works will be necessary to confirm autoimmune mechanisms in these patients (Table 1). Interestingly, many of these autoantibodies recognize cerebellar specific antigens located in Purkinje cell soma to dendrites resulting in an immunohistochemical staining pattern of ‘Medusa head’ and suggesting a common entity (see Table 1) [8–10].

**Table 1:** Representative autoantibodies to cerebellar antigens in immune-mediated cerebellar ataxias

Autoantibodies	Conditions (When autoantibodies are associated with neoplasms, frequency of PCD is indicated.)	Localization
Anti-Yo	PCD (53 %), Breast, Uterus, Ovaries	Mainly Purkinje cells and few other neurons in the molecular layer
Anti-Hu	PCD (15 %), SCLC	All neuronal nuclei and cytolpasm
Anti-Tr	PCD (5 %), Hodgkin’s disease	Purkinje cells cytoplasm and dendrites,
Anti-CV2	PCD (4 %), SCLC, thymoma	Oligodendrocytes
Anti-Ri	PCD (2 %), Breast	All neuronal nuclei
Anti-Ma2	PCD (2 %), Testicle, Lung	Nucleoli
Anti-VGCC (P/Q type)	PCD (2 %), SCLC	Purkinje cells, cytoplasm, dendrites and dot-staining of the molecular layer
Anti-SOX1	PCD (―), SCLC	Bergman glial cell nuclei
Anti-ZIC4	PCD (―), SCLC	Neuronal nuclei
PCA-2	PCD (―), SCLC	Purkinje cells, cytoplasm, dendrites and dot-staining of the molecular layer
Anti-Homer-3	^a^	Purkinje cells, cytoplasm, dendrites and dot-staining of the molecular layer
Anti-CARP VIII	^a^	Purkinje cells, cytoplasm, dendrites and dot-staining of the molecular layer
Anti-PKCγ	^a^	Purkinje cells, cytoplasm, dendrites and dot-staining of the molecular layer
Anti-Ca/ARHGAP26	^a^	Purkinje cells, cytoplasm, dendrites and dot-staining of the molecular layer
Anti-mGluR1	^a^	Medusa
Anti-Sj/ITPR1	^b^	Purkinje cells, cytoplasm, dendrites and dot-staining of the molecular layer
Anti-Nb/AP3B2	^b^	Purkinje cells, cytoplasm, dendrites and dot-staining of the molecular layer
Anti-GluRδ2	Para/post infectious	Purkinje cells, cytoplasm, dendrites and dot-staining of the molecular layer
Anti-transglutaminase 2, 6	Gluten ataxia	
Anti-GAD65	GAD Abs-CA or PCD (―)	GABAergic neurons

Frequency among PCDs was evaluated based on our consensus paper [1]. *PCD (―)* indicates low frequency.

The localization was based on the review by Jarius and Wildemann [8].

*SCLC* small cell lung carcinoma, *Medusa* Immunohistochemistry shows ‘Medusa head’ pattern in some patients.

^a^Association of neoplasms was reported only in 1–2 patients. ^b^Conditions that trigger production of Abs are unknown.

Prospective studies by Hadjivassiliou et al. [11] showed a prevalence of immune-mediated CAs of 32 % among 320 patients with sporadic ataxia (GA 27 %, PCD 3 %, and GAD Abs-CA 3 %). This suggests a higher than expected incidence of immune-mediated CAs amongst sporadic CAs. Thus, clinicians are currently required to establish the diagnosis of immune-mediated CAs (IMCAs) and to initiate immunotherapy at an early stage [1, 10].

However, there is still uncertainty regarding the type of immunotherapy that should be used for each subtype of immune-mediated CAs. This can be explained by the following two reasons. Firstly, there are no large-scale randomized studies on the optimal therapeutic strategies in IMCAs. Due to the rarity of IMCAs, there are to date no large-scale clinical studies in this field, though there are few retrospective studies and case reports. This is also confounded by the fact that in PCD, removal of the cancer may influence the immunological process causing the cerebellar damage. Secondly, treatment-induced improvement has been evaluated more or less subjectively. Some authors used the term "improvement" loosely and the extent of the "improvement" could not be assessed from the provided description of the clinical course. Although other authors quantified the effects of treatment using the International Cooperative Ataxia Rating Scale (ICARS), small increases in the score did not necessarily correlate with improvements in daily living and therefore could not be considered clinically significant changes. Furthermore fluctuations in the ataxia symptoms and signs can be influenced by other factors such as stress, fatigue etc. There is therefore a need to estimate the efficacy of immunotherapy by assessing improvement in daily living.

The aim of this paper was to propose guidelines for management of patients with IMCAs. Specifically, (1) we collected IMCAs cases described in published case reports and retrospective studies, and (2) we assessed the efficacy of various immunotherapies in terms of improvement of daily activity. Our study focused on GA, PCD, and GAD Abs-CA. First, we analyzed immunotherapies for GA and PCD, in both of which autoimmunity is triggered by known antigenic determinant (gluten and neoplasm, respectively). We also analyzed immunotherapies for GAD Abs-CA, in which there is no underlying condition that triggers autoimmunity. The main goals were to define the response of each subtype of IMCA to provide a rational therapeutic strategy for each subtype that could be tested.

### Gluten ataxia

#### What is the first line immunotherapy?

Whilst the benefits of a gluten-free diet in patients with coeliac disease and dermatitis herpetiformis have long been established [12], there are only a few studies on the effects of gluten-free diet on neurological manifestations. Most are case reports primarily concerning patients with established coeliac disease who then developed clinical neurological symptoms. Such studies described variable but overall favorable response to gluten-free diet [12]. In their systematic study on the effect of gluten-free diet in patients with GA, with or without enteropathy, Hadjivassiliou et al. [12] concluded that gluten-free diet is clinically useful in patients with GA [12]. Their study included 43 patients with GA, and 26 who adhered to the gluten-free diet showed serological evidence of elimination of antibodies (treatment group, versus 14 patients of the control group who refused the diet). In their study [12], patients of the two groups were matched at baseline for various variables (e.g., age, duration of CAs, severity of CAs). There were no significant differences in baseline performance in each ataxia test between the two groups. However, significant improvement was observed in the performance test scores (computerized finger-nose latency, hand or foot taping, Peg and quantitative Romberg’s test) and in the subjective global clinical impression scale in the treatment group compared with the control group. The improvement was apparent even after excluding patients with enteropathy. The study also provided serological evidence of elimination of anti-gliadin Abs as a confirmation of strict adherence to the diet.

The long-term effects of strict gluten-free diet were retrospectively examined in 371 patients with GA. Seventy four percent of these patients had mild ataxia (patient able to walk unaided), 16 % had moderate ataxia (patient needs walking aid/support to walk) and 10 % had severe ataxia (wheelchair bound). Strict gluten-free diet for one year (with evidence of elimination of all serological tests for gluten sensitivity) resulted in improvement of gait or stabilization in all. Improvement was most marked in the mild ataxia group. In the moderate and severe groups stabilization was the norm despite MR spectroscopic evidence of improvement of NAA/Cr of the vermis. There was good correlation between the clinical severity of the ataxia and the severity of the cerebellar atrophy. By contrast those patients who did not adopt a gluten free diet continued to progress both clinically and radiologically. Most of the patients who showed limited recovery had radiological evidence of cerebellar atrophy. These results suggest that strict-gluten free diet may improve or stabilize GA

A small, uncontrolled study looked at the effect of intravenous immunoglobulins (IVIg) in the treatment of 4 patients with GA free of enteropathy [13] (Additional file 1: Table S1). IVIg improved CAs in 3 of the 4 patients. However, these responders still showed CAs that interfered with their daily lives. Finally, the effect of intravenous methylprednisolone (mPSL) in GA has not been reported so far.

#### Prognostic factors

Despite adherence to gluten-free diet, few patients show gradual progression of CAs [14]. In general, such patients have cerebellar atrophy before diagnosis and started on the diet long after the onset of CAs. However, such cases are rare and by far the commonest cause of lack of response correlates with poor adherence to gluten-free diet [15, 16]. Other reasons include the presence of small amounts of gluten in most gluten-free products. In patients with high sensitivity to gluten, a minute amount of gluten is enough to cause cerebellar damage [15, 16]. In those patients who continue to be positive for anti-gliadin Ab despite strict adherence, wheat-free diet should be considered first before embarking on immunosuppressive therapy [16].

#### Immunotherapy for gluten-free resistant patients

Trials were reported for patients who failed to respond to gluten-free diet (though hypersensitivity to a small amount of gluten in gluten-free products could not be ruled out) [14, 17] (Additional file 1: Table S1). The MRI of these studied patients showed evident atrophy of the cerebellar vermis. Souayah et al. [14] reported three patients who were treated with IVIg whose CAs and neuropathic pain (small fiber neuropathy syndrome) were resistant to strict gluten-free diet. All three patients responded to the IVIg induction therapy. The effectiveness of such therapy was confirmed by worsening of CAs upon discontinuation of IVIg in two patients. Accordingly, these patients were treated with a maintenance dose of IVIg, which produced stabilization of clinical symptoms. Consistent with these results, Nanri et al. [17] reported a transient response to IVIg in two patients with GA. These results suggest that continuous immunosuppression is necessary in such patients. Thus, maintenance therapy with IVIg or immunosuppressants (mycophenolate, cyclosporin, cyclophosphamide, and mycophenolate mofetil) should be considered for long-term follow-up of patients who fail to respond to gluten-free diet provided that the patient is negative for all serological gluten-related antibodies [16].

#### Immunotherapies for GA subtypes

Some patients with gluten-related disorders present with myoclonus ataxia and refractory coeliac disease [18]. The myoclonus is cortical in origin and sometimes widespread. This entity is rare and 9 patients were reported among more than 600 patients with neurological manifestations of gluten-related disorders [18]. In spite of strict gluten-free diet, five of these 9 patients presented with persistent symptoms of malabsorption and evidence of on-going enteropathy on biopsies, and two patients developed enteropathy-associated lymphoma. Unlike CAs in GA, both the myoclonus and CAs respond poorly to gluten-free diet. Nevertheless, immunosuppression with mycophenolate mofetil improved to some extent both CAs and duodenal histopathological abnormalities [18]. The most disabling symptom remained the myoclonus. Anticonvulsants prevented secondary generalized seizures in all patients who had experienced seizures [18].

In conclusion, there is no convincing evidence that either mPSL or IVIg therapy is superior to gluten-free diet in GA. Based on the pathomechanism of gluten sensitivity, gluten-free diet, which can help eliminate triggering antigens, should be tried first. Strict gluten-free diet resulted in improvement or limited improvement of CAs in most cases. IVIg or immunosuppressants should be considered only if strict adherence to gluten-free diet (monitored by 6 monthly serological testing of anti-gliadin antibodies) for at least one year fails to result in any improvement in CAs or if CAs deteriorate rapidly [12].

### Paraneoplastic cerebellar degeneration

#### What is the first line of immunotherapy?

The clinical outcome of patients with PCD is usually poor, but vary according the associated autoantibody [19]. Tumor excision and physiotherapy are currently the cornerstone of the treatment. The survival time of patients who receive antitumor therapy is significantly longer than those who do not [20, 21]. Depending on the cancer type, surgery, radiotherapy or chemotherapies should be first considered in order to prevent metastasis and remove antigens that trigger immune-mediated processes [22].

The response to immunotherapy is often disappointing. Previous studies indicated that immunotherapies did not substantially improve CAs in patients with successfully treated malignancies [1, 20–23]. Furthermore, fewer than 10 % of patients with PCD respond to various immunotherapies [24]. Two long-term follow-up studies deserve discussion [22, 23]. Candler et al. [23] examined the outcome of long-term follow-up of 63 patients with paraneoplastic neurological disease, including 13 patients with PCD. These patients received various combinations of anti-cancer therapies, with or without immunotherapies (e.g., corticosteroids, intravenous immunoglobulins (IVIg), and/or plasma exchange). The median survival time of patients with PCD was 42 months (95 % CI 32–52). Three PCD patients (23.1 %) had died by last follow-up. Among the total number of patients with paraneoplastic neurological syndrome, 17 (26.9 %) died with a median survival time from onset of neurological symptoms of 43 months. Unfortunately, the cause of death (oncological or neurological) was not determined for individual cases. Neurological outcome was associated only with treatment of the tumor. In another study, Keime-Guibert et al. [22] examined the effects of immunotherapy in 16 PCD patients associated with anti-Hu (*n* = 10) and anti-Yo (*n* = 6) Abs. These patients received one to nine cycles of the combination of IVIg, intravenous methylprednisolone (mPSL), and cyclophosphamide. In their study, clinical improvement represented a change in Rankin score of at least 1 point, while stabilization represented lack of change in the same score after three courses of the combination therapy. Based on these criteria, none of the 16 patients showed improvement, while 3 of 9 patients with Rankin scale of ≤3 showed stabilization of CAs for 4, 16 and 35 months, respectively. The median survival time from beginning of immunotherapy was 10.2 months (range, 2–38) and 14 patients died during follow-up (88 %). Of these, death was cancer-related in 9 patients, neurological disease-related in 3, and unknown reason in 2 patients (suicide? or unknown). Thus, at the end of follow-up, only 2 patients were alive. Taken together, these long-term follow-up studies suggest that PCD with Hu or Yo-Abs are associated with severe CAs resistant to immunotherapy (including combination therapies).

Apart from the above long-term follow-up studies, several other retrospective studies and case reports have suggested the beneficial effects of corticosteroids and IVIg in patients with anti-Hu or anti-Yo antibodies (Additional file 2: Table S2) [25–31]. Among 9 responders, 1 patient received mPSL + cyclophosphamide, 2 patients IVIg, 1 patient IVIg + plasma exchange, 3 patients cyclophosphamide, 1 patient IVIg + oral prednisolone (PSL) + cyclophosphamide, and 1 patient IVIg + oral PSL + rituximab. The mean follow-up period ranged from 4 months to 10 years (median 33.9 months). Of these studies, Voltz [20] proposed a trial of immunotherapies after cancer treatment in patients with PCD. Specifically, the treatment regimen comprises one course of mPSL, followed 1 or 2 weeks later in case of lack of response by administration of IVIg. Lack of response to IVIg should be followed 1 or 2 weeks later by plasma exchange or cyclophosphamide.

#### Prognostic factors

There are no specific studies on the prognosis of PCD according to associated autoantibodies. However, prognosis seems to vary depending on the nature of the associated ONA. In some cases, such as PCD with Yo-Abs, cerebellar ataxia is clearly due to Purkinje cell death and neurological improvement is unlikely. The objective of treatment will be to stop the immune process resulting in neuronal death. In other cases, a neuronal blockage can be suspected before secondary neuronal death occurs and in these cases, immunomodulator treatment and tumor removal become an emergency. For example, anti-Tr/DNER-associated PCD is particularly remarkable for the improvement observed after efficient treatment of the associated lymphoma, irrespective of additional immunotherapy [32]. Furthermore, some patients with anti-Ri Ab also seem to respond well to immunotherapy [33]. This is by contrast to the poor prognosis of PCD with Yo, Hu or CV2/CRMP5-Abs suggesting different pathological mechanisms for each subtypes of paraneoplastic antibodies [34]. Finally some authors suggested that early introduction of immunotherapy could be associated with better outcome [20]. The data demonstrate the need for future prospective studies comparing patient outcomes and immunomodulator treatment responses based on associated autoantibodies [35].

### Anti-GAD Abs associated cerebellar ataxia

#### What is the first line immunotherapy?

Although various types of immunotherapies have been used in patients with GAD Abs-CA, most patients develop significant disability with poor prognosis [1, 7].

#### Short-term follow-up

Table 2 summarizes the effects of induction therapies in 20 patients with short-term follow-up [36–50] (see also Additional file 3: Table S3). Classification of patients into two types (subacute and chronic types) demonstrated good response to immunotherapy in patients with subacute type. The onset of CAs was defined as subacute when CAs reached their NADIR or required neurological assessment within the first 3 months of symptom presentation [51]. In subacute types, 4 (67 %) of the 6 tested patients showed excellent response to immunotherapy. The induction therapy included intravenous methylprednisolone (mPSL) (*n* = 2), combination of intravenous immunoglobulins (IVIg) and plasma exchange (*n* = 1), combination of IVIg and rituximab (*n* = 1), and plasma exchange followed by rituximab (*n* = 2). In contrast, in patients with the chronic type, only 9 of the tested 19 cases (42 %) showed high response to immunotherapy. The induction therapies used in these patients were mPSL (*n* = 5), combination of mPSL and IVIg (*n* = 1), combination of mPSL and plasma exchange (*n* = 2), oral prednisolone (PSL) (*n* = 1), IVIg (*n* = 6), combination of IVIg and rituximab (*n* = 2), and plasma exchange (*n* = 2). While various types of immunotherapies were used in combination to reduce the severity of CAs, this approach suggests difficulties of remission in the chronic type. Furthermore, during induction therapy, relapses occurred more frequently in the chronic type than in the subacute type. Amelioration of CAs following immunotherapy in patients with subacute and chronic types was associated with simultaneous decrease in GAD-Abs titers in most cases.

**Table 2:** Efficacies of induction therapy in 20 previously reported patients with anti-GAD antibodies associated cerebellar ataxia. Data based on short-term follow-up

	High responders	Low responders	No change	Sum
Subacute type				
mPSL	2	0	0	2
IVIg + PE	0	0	1	1
IVIg + R	1	0	0	1
PE + R	1	0	1	2
Sum	4	0	2	6
Chronic type				
mPSL	2	1	2	5
mPSL + IVIg	0	1	0	1
mPSL + PE	1	1	0	2
oral PSL	0	1	0	1
IVIg	3	1	2	6
IVIg + R	0	1	1	2
PE	2	0	0	2
Sum	8	6	5	19^a^

^a^Since two of 14 patients were repeatedly treated due to relapse, the number of therapies was 19.

*mPSL* intravenous methylprednisolone, *oral PSL* oral prednisolone, *IVIg* intravenous immunoglobulins, *PE* plasma exchange, *R* rituximab

#### Long-term follow-up

Recently, Arińo et al. [51] reported a retrospective study on the long-term effect of immunotherapy in patients with GAD Abs-CA [51]. In their study, 20 patients received immunotherapies (IVIg in 10, mPSL in 9, including mPSL alone in 4, mPSL + IVIg in 4, and mPSL + rituximab in 1, and oral PSL in 1), while 5 patients were not treated. Seventeen of the 20 treated patients received maintenance therapy, including 6 patients who received repeated IVIg during a median period of 56.2 months (24.4–121.5 months), and 11 who received one or combination of oral PSL, azathioprine, or mycophenolate mofetil. The median follow-up period was 5.4 years (3.1–10.3 years). The results can be summarized as follows. First, patients who received no immunotherapy showed progression of CAs. 2 of 5 patients showed moderately severe disabilities; they could not attend to own bodily needs without assistance, and could not walk unaided. One patient was bedridden. Second, immunotherapy improved CAs in 5 of 7 patients (71 %) patients with subacute type, similar to the results of short-term follow-up (prevalence of high response: 67 %). Three of these five patients showed no significant disability, and were able to carry out all usual activities despite some CAs. In contrast, the efficacy of immunotherapy was limited in patients with chronic type (2 of 13 patients, 15 %), which is in contrast to the literature surveys of short-term follow-up in 20 patients (proportion of patients who showed high response: 42 %). Even after immunotherapy, 3 patients still required help in daily living activities, although they were able to walk unaided, and 4 patients were unable to attend to own bodily needs without assistance, and unable to walk unaided.

Arińo et al. [51] assessed the long-term outcome of immunotherapy using modified Rankin Scale. In that study, immunotherapy was considered effective when the score improved by more than 1 point. Thus, the response to immunotherapy documented in short-term follow-up would be too small and insufficient for improvement in daily lives. Another reason for the poor response in the chronic type is the progressive nature of the chronic type. CA was progressive in 5 of the 12 patients with chronic type, suggesting that efficacies in the chronic type do not persist in the long-term. These inherent properties are different from the subacute type. In patients with subacute type, most of whom received maintenance therapies, the remaining CA was stable (4 of 6 patients), and progression was observed only in 1 of 6 patients. The third finding obtained from long-term studies is the factor responsible for the good response; patients with good outcome had a better modified Rankin Scale score at diagnosis than patients with bad outcome. Finally, Arińo et al. [51] did not recommend any particular type of immunotherapy.

In conclusion, there is no standardized immunotherapy for patients with subacute GAD Abs-CA. Induction therapies should include the combination of mPSL, IVIg, plasma exchange and rituximab to reduce CAs as much as possible, followed by maintenance therapies, such as oral PSL, azathioprine, mycophenolate mofetil, or repeated IVIg. Immunotherapy can result in long-term improvement of CAs in most patients. On the other hand, immunotherapy of patients with chronic type of GAD Abs-CA is often ineffective; it induces limited improvement in most patients, who sometimes show progression during long-term follow-up.

#### Immunotherapies for subtypes of GAD Abs-CA

Previous studies reported a group of CA patients associated with low-titers of GAD Abs (<100 U/ml) [52]. The high- and low-titer GAD Abs-CAs could be due to different pathomechanisms. In the low-titer type, 1) GAD Abs index is not necessarily beyond >1.0 (i.e., GAD Abs are not necessarily produced intrathecally), and 2) GAD-Abs titer does not necessarily decrease with improvement in CAs (i.e., changes in GAD-Abs do not correlate with improvements in CAs), which is in contrast to the high-titer type.

Previous reports showed good response to immunotherapies [52–55]. Five of the 6 reported patients so far showed response to immunotherapies (Additional file 4: Table S4). The immunotherapies used included IVIg (*n* = 2), mPSL (*n* = 1), oral PSL (*n* = 2), and combination of mPSL and oral PSL (*n* = 1). However, long-term prognosis remains uncertain. Further accumulation of patients with low-titer GAD Abs-CA is necessary for full assessment of effects of immunotherapy and the possible association with other associated autoantibodies must be carefully studied.

Finally there is some evidence of overlap between some anti-GAD associated ataxias and gluten ataxia [56]. It is therefore important to look for serological evidence of gluten sensitivity in patients with anti-GAD antibodies as gluten free diet in such cases may be a useful therapeutic intervention.

### Hashimoto’s encephalopathy

Cerebellar type of Hashimoto’s encephalopathy (HE) has been diagnosed based on the following criteria, (1) elevated anti-thyroid antibodies and (2) responsiveness to corticosteroids [1, 6, 57–62]. A series of reports have shown underlying immune-mediated mechanisms. However, its clinical entity is still controversial because of no evidence establishing a direct correlation between anti-thyroid antibodies (Abs) and ataxia given the high prevalence of anti-thyroid Abs in the normal population [1]. Consequently, there is overlapping between HE, diagnosed based on the above criteria, and other subtypes of IMCAs. Indeed, patients with GA or GAD Abs-CA often have anti-thyroid Abs [17, 52]. Pathophysiological features of patient’s CSF are also heterogeneous [63]. Taken together, further long-term systematic studies are needed to examine how steroid-responsive IMCA associated with anti-thyroid Abs (in the absence of anti-GAD and gluten antibodies) is clinically categorized.

On the other hand, there is a group of patients with anti-thyroid Abs who showed characteristically good response to immunotherapies when compared with other subtypes of IMCAs [61]. Interestingly, some patients showed full recovery. Table 3 summarizes the effects of immunotherapies (corticosteroids and intravenous immunoglobulins (IVIg)) in 13 patients reported by Matsunaga et al. [61]. Corticosteroids were administrated in 12 of these patients. Before the therapy, their CAs severely affected their daily lives. 11 patients were not able to walk unaided, though one patient walked without support but with considerable staggering, difficulties in half turn. Treatment with corticosteroids resulted in full recovery in 4 patients ("Full recovery" in Table 3), and 4 patients started to walk without support (although with irregular stepping and instability) and to look after their own affairs without help ("Improvement" in Table 3). Another four patients showed improvement of limb ataxia, though unaided walking was still difficult ("Limited improvement" in Table 3). Oral prednisolone (PSL) was used in 2 of the 12 patients while the other 10 received methylprednisolone (mPSL) intravenously first followed in 7 patients by either oral PSL, IVIg or azathioprine. There were no significant differences between oral PSL and mPSL, or between mPSL + posttherapy (PSL, IVIg or azathioprine) and mPSL alone. On the other hand, IVIg was administrated in one of the 13 patients due to diabetes mellitus though it was later switched to cyclophosphamide. This patient could not walk unaided before treatment but limb ataxia improved slightly after IVIg, though unaided walking was still impossible. The above data do not favor IVIg relative to corticosteroids but rather suggest that corticosteroids should be considered first in the treatment.

**Table 3:** Efficacies of immunotherapies in patients with Hashimoto’s encephalopathy

	Full recovery	Improvement	Limited improvement	Sum
oral PSL	1	1	0	2
mPSL	1	0	2	3
mPSL + oral PSL	2	2	1	5
mPSL + oral PSL + IS	0	0	1	1
mPSL + IVIg	0	1	0	1
IVIg + IS	0	0	1	1
Sum	4	4	5	13

Improvement: patients started to walk without support and looked after their own affairs without help. Limited recovery: Unaided walking was still impossible.

These data were based on published studies by Matsunaga et al. [58] and personal communication with the authors.

*mPSL* intravenous methylprednisolone, o*ral PSL* oral prednisolone, *IVIg* intravenous immunoglobulins, *IS* immunosuppressants

Excellent response to immunotherapy in HE correlated significantly with positivity for anti-NH2-terminal of α-enolase (NAE) Abs [61]. NAE-Abs titer decreased after the successful steroid therapy in some patients [62]. Importantly, Matsunaga et al. [61] also concluded that the response to immunotherapy in HE did not correlate with the latency between CAs onset and initiation of such therapy. Two patients with latency to treatment of 10 years responded well to corticosteroids (Patients # 2 and 9 showed full and good recovery, respectively) and both had no cerebellar atrophy on MRI. These results suggest that immune-mediated cell loss would be slow characteristically in some patients with steroid-response IMCA associated with anti-thyroid Abs, resulting in a chance of satisfactory improvement using immunomodulators.

## Conclusion

In this review, we evaluated the efficacy of each immunotherapy based on case reports and retrospective studies. When CAs are triggered by certain known antigenic stimulants, priority should be directed towards treatment of the underlying conditions. For example, gluten-free diet in GA and removal of neoplasms in PCD. In cases where CAs progress after treatment of the underlying condition, immunotherapy should follow. On the other hand, when CAs are not triggered by any other condition (e.g., in GAD Abs-CA and steroid-response IMCA associated with anti-thyroid antibodies), induction immunotherapies is provided first to minimize CAs, followed by maintenance immunotherapy to prevent relapse. Various combinations of immunotherapies have been used, ranging from IVIg, corticosteroids, immunosuppressants, plasmapheresis, and rituximab, depending on the subtype of IMCAs (Table 4).

**Table 4:** Reported first line immunotherapy for each subtype of immune-mediated cerebellar ataxias

**Gluten ataxia**
Induction and maintenance therapies: strict gluten-free diet
(In case of no improvement and negative gluten related antibodies, immunosuppressants or IVIg )
**Paraneoplastic cerebellar degeneration**
Quick removal of neoplasm must be the first objective of treatment
Induction therapy as soon as possible: mPSL, IVIg, immunosuppressants, or/and plasma exchange
Discussion according associated Abs
Maintenance therapy: continuous oral PSL, IVIg, immunosuppressants
**Anti-GAD Abs associated cerebellar ataxia**
Induction therapy: mPSL, IVIg, immunosuppressants, plasma exchange, or/and rituximab
Maintenance therapy: continuous oral PSL, IVIg, immunosuppressants, or/and rituximab

*Abs* antibodies, *mPSL* intravenous methylprednisolone, *oral PSL* oral prednisolone, *IVIg* intravenous immunoglobulins

**Fig. 1:**
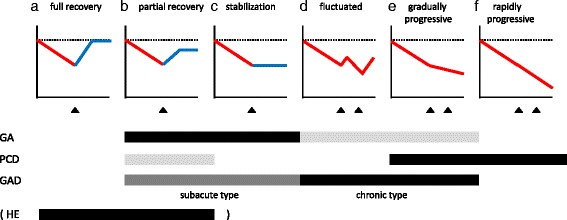
The clinical time-course could be classified into 6 patterns; **a** full recovery type, **b** partial recovery type, **c** stabilization type, **d** fluctuated type, **e** gradually progressive type, and **f** rapidly progressive type. Triangle indicates the time of immunotherapy. Possible clinical courses for each subtype are schematically indicated below. Black indicates ‘common course’, gray ‘occasional course’, and light gray ‘rare course’. *GA* gluten ataxia, *PCD* paraneoplastic cerebellar degeneration, *GAD* anti-GAD Antibodies associated cerebellar ataxia. *HE* Hashimoto’s encephalopathy, The clinical entity of HE is still controversial. Thus, we show the time-course of steroid-response IMCA associated with anti-thyroid Abs.

Previous studies suggested the presence of two distinct groups with IMCAs, the good response group, which includes GA and steroid-response IMCA associated with anti-thyroid antibodies, and poor response group, which includes GAD Abs-CA and PCD. Furthermore, the clinical time-course can be classified into 6 patterns; (a) full recovery type, (b) partial recovery type, (c) stabilization type, (d) fluctuating type, (e) gradually progressive type, and (f) rapidly progressive type (Fig. 1). These clinical courses are possibly determined by mainly two factors, responsiveness to immunotherapies and severity of cerebellar atrophy. Progression can be halted when the immune-mediated mechanisms are controlled by immunotherapy. When neural damage caused by the immune processes can be prevented by immunotherapy, the extent of cerebellar cell loss determines the recovery course, full recovery, partial recovery, or stabilization. Most patients with GA and steroid-response IMCA associated with anti-thyroid antibodies exhibit such clinical courses. In contrast, when the immune mechanism underlying CAs is resistant to immunotherapy, the clinical course would be either of fluctuated type, gradually progressive type, or progressive type. Most of patients with GAD Abs-CA and PCD show these types.

Suspicion of IMCA is the first step towards early diagnosis. There is no clear cut clinical characteristics that enable the diagnosis of IMCAs. On imaging, vermian involvement seems more prevalent in IMCAs by comparison to degenerative (MSA-C) and genetic ataxias. Rapidity of progression is a characteristic feature of PCD. In clinical practice patients presenting with ataxia without a family history should be investigated for all possible causes. IMCAs-related antibodies should be examined [1]. Even in the absence of such antibodies immune mediated ataxia may still be a possibility. The term primary autoimmune cerebellar ataxia (PACA) has been proposed to describe such entity [11]. It is likely that PACA would be equally responsive to immunomodulation. IMCAs should be diagnosed early if possible. We reported previously the preservation of cerebellar function after treatment at an early stage, prior to the development of cerebellar atrophy [1]. The development of effective therapeutic approaches then becomes essential in the treatment of IMCAs.

## Additional files

Additional file 1: Table S1. Efficacy of IVIg as induction therapy on gluten ataxia. Summary of four studies. (DOC 35 kb) Additional file 2: Table S2. Effect of immunotherapy on cerebellar ataxia with paraneoplastic cerebellar degeneration. Summary of 9 studies. (DOC 39 kb) Additional file 3: Table S3. Summary of 20 studies on the effects of various immunotherapies in patients with anti-GAD antibodies associated cerebellar ataxia. (DOC 91 kb) Additional file 4: Table S4. Summary of the effects of various immunotherapies in six patients with low-titer anti-GAD antibodies associated cerebellar ataxia. (DOC 36 kb)

## References

[1] Mitoma H, Adhikari K, Aeschlimann D, Chattopadhyay P, Hadjivassiliou M, Hampe CS (2015). Consensus Paper: Neuroimmune mechanisms of cerebellar ataxias. Cerebellum.

[2] Giommeto B, Grisold W, Vitaliani R, Graus F, Honnorat J, Bertolini G (2010). Paraneoplastic neurologic syndromes in the PNS Euronetwork database: a European study from 20 centers. Arch Neurol..

[3] Brouwer B (1919). Beitrag zur kenntnis der chronischen diffusen kleinhirnerkrankungen. Neurol Zentralbl.

[4] Ducray F, Demarquay G, Graus F, Decullier E, Antoine J-C, Giometto B (2014). Seronegative paraneoplastic cerebellar degeneration: the PNS Euronetwork experience. Eur J Neurol..

[5] Hadjivassiliou M, Grünewald RA, Chattopadhyay AK, Davies-Jones GA, Gibson A, Jarratt JA (1998). Clinical, radiological, neurophysiological and neuropathological characteristics of gluten ataxia. Lancet..

[6] Shaw PJ, Walls TJ, Newman PK, Cleland PG, Cartlidge NE (1991). Hashimoto’s encephalopathy: a steroid-responsive disorder associated with high anti-thyroid antibody titers-report of 5 cases. Neurology..

[7] Honnorat J, Saiz A, Giometto B, Vincent A, Brieva L, Andres C (2001). Cerebellar ataxia with anti-glutamic acid decarboxylase antibodies. Study of 14 patients. Arch Neurol.

[8] Jarius S, Wildemann B (2015). ‘Medusa-head ataxia’: the expanding spectrum of Purkinje cell antibodies in autoimmune cerebellar ataxia. Part 1: Anti-mGluR1, anti-Homer-3, anti-Sj/ITPR1 and anti-CARP VIII. J Neuroinflammation.

[9] Jarius S, Wildemann B (2015). ‘Medusa-head ataxia’: the expanding spectrum of Purkinje cell antibodies in autoimmune cerebellar ataxia. Part 2: Anti-PKC-gamma, anti-GluR-delta2, anti-Ca/ARHGAP26 and anti-VGCC. J Neuroinflammation.

[10] Jarius S, Wildemann B (2015). ‘Medusa-head ataxia’: the expanding spectrum of Purkinje cell antibodies in autoimmune cerebellar ataxia. Part 3: Anti-Yo/CDR2, anti-Nb/AP3B2, PCA-2, anti-Tr/DNER, other antibodies, diagnostic pitfalls, summary and outlook. J Neuroinflammation.

[11] Hadjivassiliou M, Boscolo S, Tongiorgi E, Grunewald RA, Sharrack B, Sanders DS (2008). Cerebellar ataxia as a possible organ specific autoimmune disease. Movement Disord..

[12] Hadjivassiliou M, Davies-Jones GAB, Sandres DS, Grünewald RA (2003). Dietary treatment of gluten ataxia. J Neurol Neurosurg Psychiatry..

[13] Bürk K, Melms A, Schulz JB, Dichgans J (2001). Effective of intravenous immunoglobin therapy in cerebellar ataxia associated with gluten sensitivity. Ann Neurol..

[14] Souayah N, Chin RL, Brannagan TH, Latov N, Green PHR, Kokoszka A (2008). Effect of intravenous immunoglobulin on cerebellar ataxia and neuropathic pain associated with celiac disease. Eur J Neurol..

[15] Hadjivassiliou M, Grünewald RA, Davies-Jones GAB (2002). Gluten sensitivity as a neurological illness. J Neurol Neurosurg Psychiatry..

[16] Hadjivassiliou M, Sanders DS, Woodroofe N, Williamson C, Grünewald RA (2008). Gluten ataxia. Cerebellum..

[17] Nanri K, Okita M, Takeguchi M, Taguchi T, Ishiko T, Saitjo H (2009). Intravenous immunoglobulin therapy for autoantibody-positive cerebellar ataxia. Intern Med Tokyo Jpn..

[18] Sarrigiannis PG, Hoggard N, Sanders DS, Aeschlimann D, Grunewald RA, Unwin ZC (2014). Myoclonic ataxia and refractory coeliac disease. Cerebellum & Ataxias..

[19] Demarquay G, Honnorat J (2011). Clinical presentation of immune-mediated cerebellar ataxia. Rev Neurol (Paris).

[20] Voltz R (2002). Paraneoplastic neurological syndromes: an update on diagnosis, pathogenesis, and therapy. Lancet Neurol..

[21] Dalmau J, Rosenfeld MR (2008). Paraneoplastic syndromes of the CNS. Lancet Neurol..

[22] Keime-Guibert F, Graus F, Fleury A, Renė R, Honnorat J, Broet P (2000). Treatment of paraneoplastic neurological syndromes with antineuronal antibodies (Anti-Hu, Anti-Yo) with a combination of immunoglobulins, cyclophosphamide, and methylprednisolone. J Neurol Neurosurg Psychiatry..

[23] Candler PM, Hart PE, Barnett M, Weil R, Ress JH (2004). A follow up study of patients with paraneoplastic neurological disease in the United Kingdom. J Neurol Neurosurg Psychiatry..

[24] Dropcho EJ (1998). Principles of paraneoplastic syndromes. Ann NY Acad Sci..

[25] Moll JWB, Henzen-Logmans SC, van Meche FGA, Vecht CH (1993). Early diagnosis and intravenous immune globulin therapy in paraneoplastic cerebellar degeneration. J Neurol Neurosurg Psychiatry..

[26] Stark E, Wurster U, Patzold U, Sailer M, Haas J (1995). Immunological and clinical response to immunosuppressive treatment in paraneoplastic cerebellar degeneration. Arch Neurol..

[27] Batocchi AP, Rosa GDE, Evoli A, Tonali P, Greggi S, Scambia G (1999). Positive response to therapy in a patient with seropositive paraneoplastic cerebellar degeneration and an endometrioid carcinoma of the vesicovaginal septum. J Neurol Neurosurg Psychiatry..

[28] Mowzoon N, Bradley AG (2000). Successful immunosuppressant therapy of severe progressive cerebellar degeneration and sensory neuropathy: a case report. J Neurol Sci..

[29] Phuphanich S, Brock C (2007). Neurologic improvement after high-dose intravenous immunoglobulin therapy in patients with paraneoplastic cerebellar degeneration associated with anti-Purkinje cell antibody. J Neurooncol..

[30] Thöne J, Hohaus A, Lamprecht S, Bickel A, Erbguth F (2008). Effective immunosuppressant therapy with cyclophosphamide and corticosteroids in paraneoplastic cerebellar degeneration. J Neurol Sci..

[31] Schessl J, Schuberth M, Reilich P, Schneiderat P, Strigl-Pill N, Walter MC (2011). Long-term efficiency of intravenously administered immunoglobulin in anti-Yo syndrome with paraneoplastic cerebellar degeneration. J Neurol..

[32] Briani C, Vitaliani R, Grisold W, Honnorat J, Graus F, Antoine JC (2011). Spectrum of paraneoplastic disease associated with lymphoma. Neurology..

[33] Greenlee JE (2013). Treatment of paraneoplastic cerebellar degeneration. Curr Treat Opinions Neurol..

[34] Honnorat J, Cartalat-Carel S, Ricard D, Camdessanche JP, Carpentier AF, Rogemond V (2009). Onco-neural antibodies and tumour type determine survival and neurological symptoms in paraneoplastic neurological syndromes with Hu or CV2/CRMP5 antibodies. J Neurol Neurosurg Psychiatry..

[35] Honnorat J (2014). Therapeutic approaches in antibody-associated central nervous system pathologies. Rev Neurol (Paris).

[36] Ishida K, Mitoma H, Song SY, Uchihara T, Inaba A, Eguchi S (1999). Selective suppression of cerebellar GABAergic transmission by an autoantibody to glutamic acid decarboxylase. Ann Neurol..

[37] Lauria G, Pareyson D, Pitzolu G, Bazzigaluppi E (2003). Excellent response to steroid treatment in anti-GAD cerebellar ataxia. Lancet Neurol..

[38] Birand B, Cabre P, Bonnan M, Olindo S, Smadja D (2006). A new case of cerebellar ataxia with anti-GAD antibodies treated with corticosteroids and initially seronegative. Rev Med Interne..

[39] McFarland NR, Login IS, Verno S, Burns TM (2006). Improvement with corticosteroids and azathioprine in GAD65-associated cerebellar ataxia. Neurology..

[40] Kim JY, Chung EJ, Kim JH, Jung KY, Lee WY (2006). ReVaninsponse to steroid treatment in anti-glutamic acid decarboxylase antibody-associated cerebellar ataxia, stiff-person syndrome and polyendocrinopathy. Mov Disord..

[41] Vulliemoz S, Vanini G, Truffert A, Chizzolini C, Seeck M (2007). Epilepsy and cerebellar ataxia associated with anti-glutamic acid decarboxylase antibodies. J Neurol Neurosurg Psychiatry..

[42] Chang CC, Eggers SD, Johnson JK, Haman A, Miller BL, Geschwind MD (2007). Anti-GAD antibody cerebellar ataxia mimicking Creutzfeldt-Jakob disease. Clin Neurol Neurosurg..

[43] Bonnan M, Cabre P, Olindo S, Signate A, Saint-Vil M, Smadja D (2008). Steroid treatment in four cases of anti-GAD cerebellar ataxia. Rev Neurol..

[44] Abele M, Weller M, Mescheriakov S, Bürk K, Dichgans J, Klockgether T (1999). Cerebellar ataxia with glutamic acid decarboxylase autoantibodies. Neurology..

[45] Takenoshita H, Shizuka-Ikeda M, Mitoma H, Song SY, Harigaya Y, Igeta Y (2001). Presynaptic inhibition of cerebellar GABAergic transmission by glutamate decarboxylase autoantibodies in progressive cerebellar ataxia. J Neurol Neurosurg Psychiatry..

[46] Rüegg S, Stahl M, Bühlmann M, Dupont A, Lyrer PA, Humbel RL (2002). Cerebellar degeneration and polyglandular autoimmune syndrome with anti-glutamic acid decarboxylase antibodies. J Neurol..

[47] Matsumoto S, Kusuhara T, Nakajima M, Ouma S, Takahashi M, Yamada T (2002). Acute attacks and brain stem signs in a patient with glutamic acid decarboxylase autoantibodies. J Neurol Neurosurg Psychiatry..

[48] Georgieva Z, Parton M (2014). Cerebellar ataxia and epilepsy with anti-GAD antibodies: treatment with IVIG and plasmapheresis. BMJ Case Rep.

[49] Planche V, Marques A, Ulla M, Ruivard M, Durif F (2014). Intravenous immunoglobulin and rituximab for cerebellar ataxia with glutamic acid decarboxylase autoantibodies. Cerebellum..

[50] Kuchling J, Shababi-Klein J, Nümann A, Gerischer LM, Harms L, Prüss H (2014). GAD antibody-associated late-onset cerebellar ataxia in two female siblings. Case Rep Neurol.

[51] Arińo H, Gresa-Arribas N, Blanco Y, Martínez-Hernández E, Sabater L, Petit-Pedrol M (2014). Cerebellar ataxia and glutamic acid decarboxylase antibodies. Immunologic profile and long-term effect of immunotherapy. JAMA Neurol.

[52] Nanri K, Niwa H, Mitoma H, Takei A, Ikeda J, Harada T (2013). Low-titer anti-GAD-antibody-positive cerebellar ataxia. Cerebellum..

[53] Virgilio R, Corti S, Agazzi P, Santoro D, Lanfranconi S, Candelise L (2009). Effect of steroid treatment in cerebellar ataxia associated with anti-glutamic acid decarboxylase antibodies. J Neurol Neurosurg Psychiatry..

[54] Nociti V, Frisullo G, Tartaglione T, Patanella AK, Iorio R, Tonali PA (2010). Refractory generalized seizures and cerebellar ataxia associated with anti-GAD antibodies responsive to immunosuppressive treatment. Eur J Neurol..

[55] Pedroso JL, Braga-Neto P, Dutra LA, Barsottini OGP (2011). Cerebellar ataxia associated to anti-glutamic acid decarboxylase autoantibody (anti GAD). Arq Neuropsiquiatr..

[56] Hadjivassiliou M, Aeschlimann D, Grunewald RA, Sanders DS, Sharrack B, Woodroofe N (2011). GAD antibody associated neurological illness and its relationship to gluten sensitivity. Acta Neurol Scand..

[57] Manto M, Goldman S, Bodur H (1996). Cerebellar syndrome associated with Hashimoto’s encephalopathy. Rev Neurol (Paris).

[58] Selim M, Drachman DA (2001). Ataxia associated with Hashimoto’s disease: progressive non-familial adult onset cerebellar degeneration with autoimmune thyroiditis. J Neurol Neurosurg Psychiatry..

[59] Passarella B, Negro C, Nozzoli C, De Marco V, Rini A (2005). Cerebellar subacute syndrome due to corticosteroid-responsive encephalopathy associated with autoimmune thyroiditis (also called ‘Hashimoto’s encephalopathy’). Clin Ter..

[60] Tang Y, Chu C, Lin MT, Wei G, Zhang X, Da Y (2011). Hashimoto’s encephalopathy mimicking spinocerebellar ataxia. J Neurol..

[61] Matsunaga A, Ikawa M, Fujii A, Nakamoto Y, Kuriyama M, Yoneda M (2013). Hashimoto’s encephalopathy as a treatable adult-onset cerebellar ataxia mimicking spinocerebellar degeneration. Eur Neurol..

[62] Nakagawa H, Yoneda M, Fujii A, Kinomoto K, Kuriyama M (2007). Hashimoto’s encephalopathy presenting with progressive cerebellar ataxia. J Neurol Neurosurg Psychiatry..

[63] Mitoma H, Yoneda M, Saitow F, Suzuki H, Matsunaga A, Ikawa M (2014). Presynaptic dysfunction caused by cerebrospinal fluid from a patient with the ataxic form of Hashimoto’s encephalopathy. Neurol Clin Neurosci.

