# Supplementary material for: Guidelines for treatment of immune-mediated cerebellar ataxias
Source: Cerebellum Ataxias. 2015 Nov 10;2:14. doi: 10.1186/s40673-015-0034-y (PMC4641375; doi:10.1186/s40673-015-0034-y)
Supplement: Additional file 1: Table S1. — Efficacy of IVIg as induction therapy on gluten ataxia. Summary of four studies. (DOC 35 kb) [file 40673_2015_34_MOESM1_ESM.doc]

Additional file 1: Table S1. Efficacy of IVIg as induction therapy on gluten ataxia. Summary of four studies.

| Age/Gender | Delay  MRI | Outcome of induction therapy | Outcome of maintenance therapy |
| --- | --- | --- | --- |
| Bürk et al. (2001) [✳ ICARS is estimated based on the figure] | | |  |
| 33/F | Unknown.  No villous atrophy | IVIg (0.4g/kg/day) for 5 days  ICARS: 42→22✳ | Unknown |
| 66/M | Unknown.  No villous atrophy | IVIg (0.4g/kg/day) for 5 days.  ICARS: 30→9✳ | Unknown |
| 68/M | Unknown.  No villous atrophy | IVIg (0.4g/kg/day) for 5 days.  ICARS: 42→32✳ | Unknown |
| 65/M | Unknown.  No villous atrophy | IVIg (0.4g/kg/day) for 5 days.  ICARS: 21→21✳ | Unknown |
| Efficacy of IVIg in patients with resistance to strict gluten-free diet (n=5) | | | |
| Souayah et al. (2008) | |  |  |
| 37/F | 12 years  Atrophy | IVIg (2g/kg)  ICARS: 31→3.  Relapse 5 weeks later.  ICARS: 17 | IVIg (0.5-0.75 g/kg).  2-4 weeks.  ICARS: 3 to 13 |
| 41/F | 3 years  Not described | IVIg (2g/kg, 4 doses for 2 wks).  ICARS: 10→NA | IVIg (60 g) every 2 wks for 3 months  ICARS: 0  Relapse 3 months after suspension |
| 42/F | 2 years  Mild atrophy | IVIg (2 g/kg, 4 doses for 2 wks)  ICARS: 16→NA | IVIg (40 g) every 2 wks for 3 months  ICARS: 7  IVIg (50g)  Every 2 wks for 20 months  ICARS: 3 |
| Nanri et al. (2009) | |  |  |
| 84/F | 7 years  Atrophy | IVIg (0.4 g/kg/day for 4 days) ICARS: 31→22 | Unknown |
| 51/M | 5 years  Atrophy | IVIg (0.4g/kg/ day for 4 days) ICARS: 19→5 | Unknown |

IVIg; intravenous immunoglobulins
