# Supplementary material for: Guidelines for treatment of immune-mediated cerebellar ataxias
Source: Cerebellum Ataxias. 2015 Nov 10;2:14. doi: 10.1186/s40673-015-0034-y (PMC4641375; doi:10.1186/s40673-015-0034-y)
Supplement: Additional file 2: Table S2. — Effect of immunotherapy on cerebellar ataxia with paraneoplastic cerebellar degeneration. Summary of 9 studies. (DOC 39 kb) [file 40673_2015_34_MOESM2_ESM.doc]

Additional file 2: Table S2. Effect of immunotherapy on cerebellar ataxia with paraneoplastic cerebellar degeneration. Summary of 9 studies.

| Age/Gender | Associated neoplasm  Treatment | Delay | Autoantibodies  MRI | Immunotherapy | Outcome.  Follow-up period (months) |
| --- | --- | --- | --- | --- | --- |
| Moll et al. (1993) |  |  |  |  |  |
| 44/F | Breast cancer.  Surgery, Chemotherapy | 13 days | No identified Abs.  No atrophy | Plasmapheresis  IVIg | Gait with assistance → gait without assistance.  8 |
| Stark et al. (1995) |  |  |  |  |  |
| 61/F | Ovarian tube cancer.  Surgery, chemotherapy | 2.5 months | Not assessed.  Mild atrophy | oral PSL (no effect), CS, CP | Gait with assistance → gait with wheel chair.  54 |
| 51/F | Ovarian cancer.  Surgery | 24 days | Not assessed.  ND | CP | Moderate ataxic gait →minimal ataxic gait.  24 |
| Batocchi et al. (1999) |  |  |  |  |  |
| 47/F | Pelvic endometroid cancer. Surgery, Chemoradiotherapy | 6 months | Anti-Yo Ab.  Mild atrophy | CP | ? → gait with wheel chair.  21 |
| Mowzoon & Bradley (2000) |  |  |  |  |  |
| 56/F | Unknown | 19 months | Negative.  Mild atrophy | IVIg, oral PSL, CP | Gait with wheel chair →gait with cane.  18 |
| Phuphanich & Brock (2007) |  |  |  |  |  |
| 72/F | Papillary carcinoma (extraovarian origin)  Chemotherapy | 12 months | Anti-Yo Ab.  Atrophy | IVIg | ? → able to swallow.  18 |
| 54/F | Ovarian cancer.  Surgery, chemotherapy | 6 months | Anti-Yo Ab.  ND | IVIg | Gait with wheel chair →gait with assistance.  ND |
| Thöne et al. (2008) |  |  |  |  |  |
| 86F | Ovarian cancer | 5 months | Anti-Yo Ab.  Normal | mPSL, oral PSL, CP | Gait wit assistance →gait with walking frame.  4 |
| Schessl et al. (2011) |  |  |  |  |  |
| 72/F | Ovarian cancer.  Surgery, chemotherapy | ND | Anti Yo Ab.  Normal | IVIg, oral PSL, rituximab | Gait with assistance decrease in falling.  120 |

mPSL; intravenous methylprednisolone, oral PSL; oral prednisolone, IVIg; intravenous immunoglobulins, CS: Cyclosporine, CP: Cyclophosphamide, ND: Not described
