# Supplementary material for: Guidelines for treatment of immune-mediated cerebellar ataxias
Source: Cerebellum Ataxias. 2015 Nov 10;2:14. doi: 10.1186/s40673-015-0034-y (PMC4641375; doi:10.1186/s40673-015-0034-y)
Supplement: Additional file 3: Table S3. — Summary of 20 studies on the effects of various immunotherapies in patients with anti-GAD antibodies associated cerebellar ataxia. (DOC 91 kb) [file 40673_2015_34_MOESM3_ESM.doc]

Additional file 3: Table S3. Summary of 20 studies on the effects of various immunotherapies in patients with anti-GAD antibodies associated cerebellar ataxia.

| Age/Gender | Delay Subtype | GAD Abs  MRI | Induction / maintenance therapy | Outcome  Estimation | GAD Abs |
| --- | --- | --- | --- | --- | --- |
| **Corticosteroids** |  |  |  |  |  |
| Ishida et al. (1998) |  |  |  |  |  |
| 66/F | 7 months.  Chronic | 77,000 U/ml.  Atrophy | oral PSL / oral PSL, plasma exchange | Ataxias: slight recovery  Low response  Subsequent progression of ataxia. Death 4 years after first therapy. | Decreased |
| Lauria et al. (2003) |  |  |  |  |  |
| 66/F | 5 months  Chronic | 531,000 U/l  Mild atrophy | [First therapy] mPSL+IVIg / oral PSL  [Second therapy] mPSL / oral PSL+CP | ICARS: 60→10  Low response (relapsed after 12 months)  ICARS: 36→8  High response (for 6 months) | Decreased  Decreased |
| Birand et al. (2006) |  |  |  |  |  |
| 38/F | 33 months Chronic? | 6,472 U/ml  Atrophy | mPSL / none | ICARS: 61→51  Low response (18 months) |  |
| McFarland et al. (2006) |  |  |  |  |  |
| 70/M | 9 months  Chronic | 10,018 U/ml  Normal | 1) mPSL  2) Plasma exchange  3) mPSL  / azathioprine | ICARS: 78→20 (relapse after 2 months)  ICARS: 29→30  ICARS: 30→6  High response (for 12 months from azathioprine medication) | Decreased Decreased Decreased |
| Kim et al. (2006) |  |  |  |  |  |
| 40/F  +Stiff person syndrome | 4 months  Chronic | 92,680 U/ml  ND | 1) mPSL  2) mPSL  / oral PSL | ICARS: 31→20 (relapse after 4 months)  ICARS: 23→11  High response (for 5 month) | Decreased Decreased |
| Vulliemoz et al. (2007) |  |  |  |  |  |
| 58/M  +Epilepsy | 2 months Subacute | 1/8000  Normal | mPSL /oral PSL, azathioprine | Gait with aid→gait without aid  High response (for 8 months) | ND |
| Chang et al. (2007) |  |  |  |  |  |
| 56/F  +Cognition failure | 3 months Subacute | 1,752 nmol/l Normal | mPSL / oral PSL, plasma exchange  + plasma exchange  + mPSL (monthly for 9 months)  + mycophenolate mofefill | Gait with aid→gait without aid  High response (for 15 months) | Decreased |
| Bonnan et al. (2008) |  |  |  |  |  |
| 38/F | 12 months Chronic | >30,000 U/ml  ND | mPSL × 6+plasma exchange / none | ICARS: 60→52  Low response (36 months) | Fluctuated |
| 45/F | 12 months  Chronic | 9,565 U/ml  ND | mPS L × 6 / azathioprine+ periodic IVIg | ICARS: 8→12  Progression (31 months) | Fluctuated |
| 75/F | 10 months Chronic | 302/U/ml  ND | mPSL × 6 / none | ICARS: 16→14  No change (12 months) | Fluctuated |

| Age/Gender | Delay Subtype | GAD Abs  MRI | Induction / maintenance therapy | Outcome  Estimation | GAD Abs |
| --- | --- | --- | --- | --- | --- |
| **IVIg** |  |  |  |  |  |
| Abele et al. (1999) |  |  |  |  |  |
| 68/F | 18 years  Chronic | >1,000(U/l)  Atrophy | 1) IVIg  2) IVIg  / none | ICARS: 59→50  ICACRS: 50→48  Low response (for 3 months) | No change |
| Takenoshita et al. (2001) |  |  |  |  |  |
| 72/F | 2 years  Chronic | 95 500 (U/ml)  ND | IVIg / none | Ataxias: unchanged  No change (for 3 months**)** | No change |
| Rüegg et al. (2002) |  |  |  |  |  |
| 62/F | 1 year  Chronic | 321 (U/l)  Mild atrophy | IVIg / none | Gait ataxia: unchanged  No change (for 1 month) | ND |
| Matsumoto et al. (2002) |  |  |  |  |  |
| 63/F | 1 month  Subacute | 10,400 (U/ml)  Normal | 1) Plasmapheresis  2) IVIg  / none | Gait with aid→gait without aid (relapsed after 3 weeks)  Prominent gait ataxia: unchanged  No change (for 1 month) | Decreased |
| Georgieva and Parton (2014) |  |  |  |  |  |
| 45/M  +epilepsy | 7 years  Chronic | Strongly positive  Mild atrophy | [First therapy] IVIg / azathioprine  [Second therapy] IVIg / azathioprine  [Third therapy] Plasma exchange  / azathioprin  [Fourth therapy] IVIg / azathioprine  [Fifth therapy] Plasma exchange  / azathioprin | ICARS: 8→4.5  High response (relapsed after 10 weeks)  ICARS: 7.5→5.5  High response (relapsed after 10 weeks)  ICARS 15→6  High response (relapsed after 12 weeks)  ICARS: 12.5→10  High response (relapsed after 8 weeks)  ICARS 12→8  High response (relapsed after 8 weeks) | ND |
| Planche et al. (2014) |  |  |  |  |  |
| 72/F | 6 months  Subacute | >250 (U/ml)  Normal | 1) IVIg×2  2) Rituximab  / none | Ataxias: progressive  ICARS: 22→10  High response (for 14months) | Increased  Decreased |
| 73/F | 3 years  Chronic? | >221 (U/ml)  Norma | 1) IVIg×2  2) Rituximab  / IVIg×3 | Ataxias: slight recovery  ICARS: 22→21  Low response (for 17 months) | ND  No change |
| 65/M | 3 years  Chronic? | >1,000 (U/ml)  Atrophy | 1) IVIg  2) IVIg+rituximab+CP  / none | Ataxias: progressive  ICARS: 35→43  Progressed (for 16 months) | No change  No change |

| Age/Gender | Delay Subtype | GAD Abs  MRI | Induction / maintenance therapy | Outcome  Estimation | GAD Abs |
| --- | --- | --- | --- | --- | --- |
| **Plasmapheresis**  **+rituximab** |  |  |  |  |  |
| Kuchhhling et al. (2014) |  |  |  |  |  |
| 74/F | 6 months  Subacute | > 2,000 IU/ml  Normal | immunoadsorption+ rituximab / none | gait with aid→gait without aid  High response (for 1 month) | ND |
| 76/F | 6 years  Subacute | > 2,000 IU/ml  Normal | immunoadsorption+ rituximab / none | Prominent gait ataxia: unchanged  No change (for 1 month) | ND |

mPSL; intravenous methylprednisolone, oral PSL; oral prednisolone, IVIg; intravenous immunoglobulins, CP: Cyclophosphamide, R; ritzuximab, ND: Not described
