# Supplementary material for: Guidelines for treatment of immune-mediated cerebellar ataxias
Source: Cerebellum Ataxias. 2015 Nov 10;2:14. doi: 10.1186/s40673-015-0034-y (PMC4641375; doi:10.1186/s40673-015-0034-y)
Supplement: Additional file 4: Table S4. — Summary of the effects of various immunotherapies in six patients with low-titer anti-GAD antibodies associated cerebellar ataxia. (DOC 36 kb) [file 40673_2015_34_MOESM4_ESM.doc]

Additional file 4: Table S4. Summary of the effects of various immunotherapies in six patients with low-titer anti-GAD antibodies associated cerebellar ataxia.

| Age/Gender | GAD Abs | Induction | Outcome |
| --- | --- | --- | --- |
|  | MRI | /Maintenance therapy | Estimation |
| Virgilio et al. (2009) |  |  |  |
| 76/F | 14.6 (U/ml) | mPSL | ICARS: 38→7 |
|  | / unknown | High response |  |
| Nocti et al. (2010) |  |  |  |
| 42/F | 67.4 (U/ml) | oral PSL+azathioprone | marked improvement |
|  | Mild atrophy | / unknown | High response |
| Pedroso et al. (2011) |  |  |  |
| 51/M | 3.2 (U/ml) | IVIg | ICARs: 65→32 |
|  | Mild atrophy | / unknown | High response |
| Nanri et al. (2013) |  |  |  |
| 52/F | 3.0 (U/ml) | IVIg | ICARS: 46→35 |
|  | Atrophy | / unknown | High response |
| 51/F | 28.6 (U/ml) | oral PSL | ICARS: 27→14 |
|  | Atrophy | / unknown | High response |
| 52/F | 6.7 (U/ml) | mPSL+oral PSL | only transient response |
|  | Atrophy | / unknown | Low response |

mPSL; intravenous methylprednisolone , oral PSL; oral prednisolone, IVIg; intravenous immunoglobulins
